# Vector field analysis for surface registration in computer‐assisted ENT surgery

**DOI:** 10.1002/rcs.1977

**Published:** 2019-01-07

**Authors:** Georgi Diakov, Wolfgang Freysinger

**Affiliations:** ^1^ Department of Oto‐, Rhino‐, Laryngology 4D‐Visualization Laboratory, Innsbruck Medical University Innsbruck Austria

## Abstract

**Background:**

Manual paired‐point registration for navigated ENT‐surgery is prone to human errors; automatic surface registration is often caught in local minima.

**Methods:**

Anatomical features of the human occiput are integrated into an algorithm for surface registration. A vector force field is defined between the patient and operating room datasets; registration is facilitated through gradient‐based vector field analysis optimization of an energy function. The method is validated exemplarily on patient surface data provided by a mechanically positioned A‐mode ultrasound sensor.

**Results:**

Successful registrations were achieved within the entire parameter space, as well as from positions of local minima that were found by the Gaussian fields algorithm for surface registration. Sub‐millimetric registration error was measured in clinically relevant anatomical areas on the anterior skull and within the generally accepted margin of 1.5 mm for the entire head.

**Conclusion:**

The satisfactory behavior of this approach potentially suggests a wider clinical integration.

## 1 INTRODUCTION

### 1.1 Motivation

Manual paired‐point registration for navigated ears, nose, and throat (ENT) surgery is prone to human errors localizing registration markers in the diagnostic images and on the patient,^1^, ^2^, ^3^, ^4^ respectively. The procedure can be automated through surface registration with intraoperatively acquired data; however, the state‐of‐the‐art algorithms either converge locally,^5^, ^6^, ^7^, ^8^, ^9^, ^10^, ^11^, ^12^, ^13^, ^14^ or rely on expensive brute force computation.^15^

Various methods for intraoperative acquisition of patient surface data for registration are limited by requirements for a direct line‐of‐sight,^16^ maintenance of a constant angle of incidence^17^, ^18^, ^19^, ^20^, ^21^, ^22^, ^23^, ^24^, ^25^ of the scanning beam, susceptibility to the illumination conditions,^26^, ^27^, ^28^, ^29^, ^30^ and anaesthetization status of the patient.^18^

### 1.2 Brief overview of surface registration methods

Surface registration is increasingly being used in clinical applications and state‐of‐the‐art navigation systems by BrainLab (Munich, Germany) and Medtronic (Minneapolis, MN, USA), such as StealthStation S7. The iterative closest point (ICP) algorithm and its variants^5^, ^6^, ^7^, ^8^, ^9^, ^10^, ^11^, ^12^, ^13^, ^14^ are the generically used approaches. However, they do not guarantee convergence to the global minimum of the cost function. The error metric^9^, ^31^ to be minimized by the optimizer^9^, ^32^, ^33^ is formulated as the root‐mean‐square of the closest distances. In parameter space, defined by Euclidean coordinates of the surface points, a guaranteed closed‐form solution^34^ cannot be found.^5^

A variety of approaches have been suggested to solve the issue of convergence of the ICP algorithm to local minima. The integration of probabilistic point weights^10^ and geometrical features^12^, ^13^ into the cost function outperformed ICP in registering computer tomography (CT) to A‐mode ultrasound and magnetic resonance data, respectively. However, convergence still needs a rough initialization. Improved convergence rates on the human femur and robustness to noise were achieved by a probabilistic variation of ICP, incorporating both positional and orientational information.^35^ A combination of principal‐axes‐based registration and Hausdorff distance minimization^36^ was used for automated matching of electro‐anatomical and CT data, applicable on closed surfaces only. Manually delineated salient anatomical features^14^ lead to lower target registration error (TRE)^37^, ^38^ than ICP; however, additional tuning of the anatomical features was required.

Additional information to ICP is usually coded as scalar attributes, or covariance matrices,^11^ accounting for the anisotropy of localized surface points. Minimization of anisotropically weighted distances within points' Voronoi regions leads to improved convergence rates and better accuracy than the classical ICP, even in the presence of noise in time‐of‐flight (ToF) range data; results are highly sensitive to the choice of covariance matrices.

The coherent point drift^39^ is a probabilistic approach to surface registration, implying coherent motion of the centroids of Gaussian mixture models. It generalizes well to non‐rigid registration and outperforms ICP for brain shift estimation.^40^ For rigid registration, however, pre‐alignment of the datasets is still necessary, and the basin of convergence is limited to ±70°.

The GoICP algorithm^15^ achieves global convergence and is robust against outliers. Splitting the parameter space into a branching and bounding scheme, nesting, and trimming of unpromising subspaces still results in a large global domain to be searched for minima of the cost function. GoICP relies on brute force computation, and registration can be very time intensive.

The Gaussian fields (GF) method for surface registration^41^ encodes local shape information through form attributes in the formulation of the cost function. Gaussian convolution^31^ achieves differentiability and convexity in the neighborhood of the aligned position. The basin of convergence is further extended (though to a certain limit) through relaxation of the Gaussian aperture, resulting in a higher residual error. Global convergence of GF cannot be guaranteed and depends on proper detection and weighting of the form attributes in the cost function.

### 1.3 Brief overview of intraoperative acquisition methods

Various modalities have been used to acquire intraoperative surface data with an optically tracked hand‐held probe, such as a mechanical pointer,^16^ or ultrasound.^17^, ^18^, ^19^, ^20^, ^21^, ^22^, ^23^, ^24^, ^25^ More advanced technologies, like positron imaging^42^ or conoscopic holography,^43^ achieve improved quality of the data and automatic removal of imaging artifacts. Common drawbacks by these acquisition methods are the requirements for an uninterrupted direct line‐of‐sight and maintenance of a constant angle of incidence of the scanning beam.

Laser acquisition of the skin surface^26^, ^27^, ^28^ is prone to deviations from the pre‐operatively generated model, due to skin elasticity. Intraoperative in‐situ laser scanning of cartilage surface achieves high precision and accuracy^29^; however, it is susceptible to stray‐light. The localization error is strongly influenced by the angle of incidence and increases at greater depths.

ToF cameras represent a novel method, still under investigation, allowing fast and robust distance measurements on the patient.^30^ Its application is still hindered by sensitivity to background light, reflections, and interference between multiple ToF devices. Hybrid methods, combining several modalities, are applied for tracking of inaccessible anatomical areas.^44^

Registration of a 3D‐model from multi‐view stereo reconstructions of the facial relief^45^ resulted in clinically relevant accuracy in robotic neurosurgery. The use of curvatures for the detection of geometric features in the generated models is based on a well‐developed theory, using differential geometry. The method can be tracked back to early studies,^46^ reporting registration of a CT‐segmentation to traces of points, intraoperatively acquired with ultrasound. Curvature‐based features in stereo reconstructions from range and ToF images were detected and described through differential geometry and B‐spline approximation for an improved accuracy and temporal stability in image‐guided radiation therapy.^47^

Most of the drawbacks of acquisition methods, eg, changes of patient's anatomy, due to skin‐shifts after anaesthetization, can be overcome by A‐mode ultrasound,^17^, ^18^, ^19^, ^20^, ^21^, ^22^, ^23^, ^24^, ^25^ enabling intraoperative scanning of the bone surface and thus a rigid body registration, characterized by the highest clinical accuracy. The method is especially suited for navigated surgery of the head, where the relevant anatomy is confined within the skull. The ultrasound beam propagates through soft tissue and thus eliminates the requirement for surgical exposure of the scanned bone surface. As mentioned above, the main challenges by this approach come out of the drawbacks of the optical tracking of a hand‐held ultrasound probe.

### 1.4 Basis outline of the paper

1. In this contribution a major step towards global convergence of surface registration has been made. A binary energy function is minimized, introducing novelty methods, such as:
  - Instant center of rotation in the parameterization of the energy function;
  - Vector field analysis (VFA) for the detection of characteristic points in the optimization.
  As to our knowledge, the above‐mentioned techniques are unique and unprecedented in the existing literature.
2. The surface registration algorithm was validated on a skull phantom and a test bed, developed for the specific application in navigated ENT‐surgery. Intraoperative data acquisition was automated with A‐mode ultrasound in the context of registration on the posterior skull.
3. An intuitive tool was developed for visualization of the TRE on a color‐coded distance map.

## 2 MATERIALS AND METHODS

### 2.1 Laboratory setup

**Figure 1:**
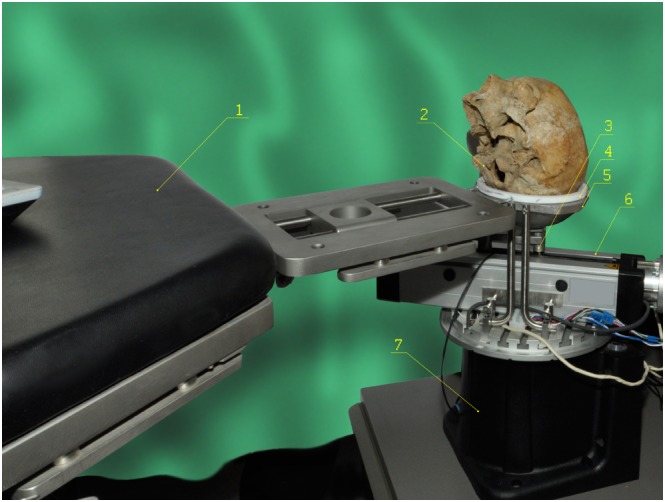
Operating table with attached registration device. The A‐mode sensor is positioned with electro‐mechanical modules for rotation and translation. Phantom's head is accommodated on a hemispherical PTFE shell. Description: 1. Operating table, 2. Skull phantom, 3. Ultrasound sensor, 4. PTFE shell, 5. Stainless steel shell (with a channel cut for the sensor), 6. Module for translation, 7. Module for rotation

A laboratory setup was built for validation of surface registration methods in navigated ENT‐surgery (Figure 1). A skull phantom is accommodated on a hemispherical shell, made of polytetrafluoroethylene (PTFE), and mounted to the operating table. The methods for scanning of the occiput with a mechanically positioned A‐mode ultrasound sensor, the signal processing, and the generation of a surface model are described in Diakov et al.^48^ Along the propagation path, the signal is transmitted through media of different acoustic impedances^9^ (PTFE, gel, and bone). At acoustic interfaces, it is partially reflected and partially transmitted, depending on the difference between the acoustic impedances. The strongest echo is at the interface gel‐bone, where the speed of sound (and thus the directly proportional acoustic impedance) alters from 1500 to 3600 m/s and the signal is almost fully reflected.

**Figure 2:**
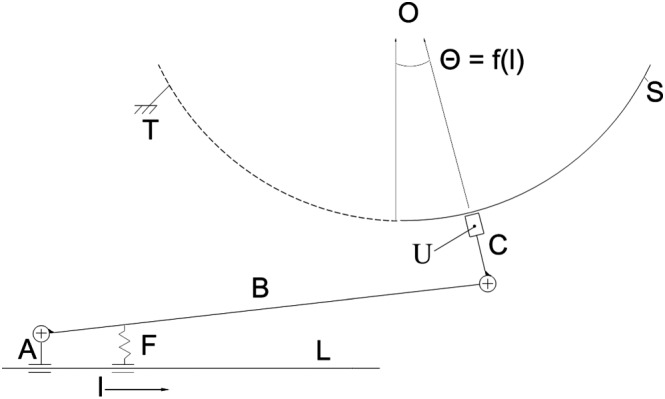
Kinematic scheme of the experimental setup. The sledge (A) is positioned by the translation module along the linear path (L). The crankshaft (B) is connected to (A) through a cylindrical joint. Another cylindrical joint connects (B) to the sensor holder (C). The active surface of the ultrasound sensor (U) slides on the surface of the hemispherical PTFE shell (S).The constant contact of the active element with the PTFE shell is secured through the elastic spring (F), which is fixed to (A). The ultrasound beam propagates radially to the center (O) of the PTFE shell and reflects from the surface of the posterior skull. The angle Θ is determined from the translation distance (l) of the sledge (A). The module for translation (L) is fixed on the module for rotation (not shown), which rotates the whole setup around the vertical axis (in the figure), while the PTFE shell (S) remains fixed to the operating table (T). Thus, by adjusting of the angles Θ and Φ (angle of the module for rotation), U can reach any 3D‐point on the hemi‐spherical surface of S. The hemispherical trajectory of the sensor is a first approximation of the occipital bone relief

A polar coordinate system is defined with an origin at the center of the hemispherical PTFE shell. The angle of rotation is the Φ polar coordinate, while the translation of the sensor is trigonometrically transformed into the Θ polar coordinate (Figure 2). The polar radius is computed from the ToF of the ultrasound waves. A 3D mesh is built from the ultrasound echoes, where the sampling takes 0.125 seconds per spatial point.

**Figure 3:**
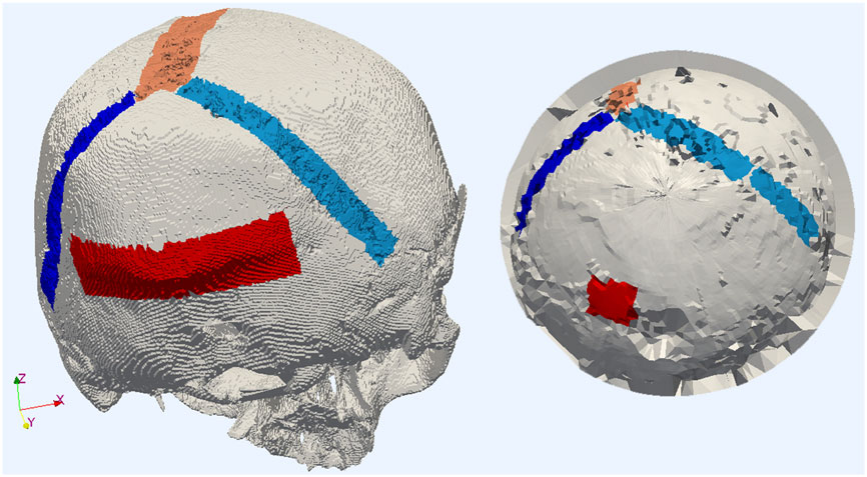
Datasets for surface registration. Left: 3D‐model from a segmentation of a CT‐image. Right: 3D‐model from an A‐mode ultrasound scan. The anatomical features in both models are marked with form attributes. The color differs for each suture of the Lambda fissure and the protuberantia occipitalis externa. (visualization: ParaView)

A preoperative diagnostic CT‐image (Siemens Sensation 16, Siemens Healthcare GmbH, Erlangen, Germany, voxel dimensions 0.42 × 0.42 × 0.6 mm) of a skull phantom was segmented through a gray‐value filter in the visualization toolkit (VTK, ver. 7.1, Kitware Inc., Clifton Park, New York, USA), and a 3D‐surface model was generated in 3D‐Slicer (ver. 4.3, Massachusetts Institute of Technology and Brigham and Women's Hospital, MA, USA) through marching cubes,^49^ resulting in approximately 680 000 spatial points. The posterior skull was scanned with a mechanically positioned ultrasound sensor (Figure 1), and the bone surface was reconstructed from the scan. The ultrasound sensor was positioned in the polar coordinate range of [0°; 360°] (Φ‐polar coordinate) and [−60°; 60°] (Θ‐polar coordinate), with an angular resolution of one degree. Figure 3 shows the 3D‐models, generated from CT‐data (left) and from the A‐mode ultrasound acquisition (right). The characteristic anatomical relief features (the Lambda fissure and protuberantia occipitalis externa) are manually marked with form attributes.

### 2.2 Surface registration through vector field analysis

The anatomical structures of the posterior skull (Figure 3) were utilized for surface registration. Unique form attributes were assigned to the 3D points, belonging to each of the sutures of the Lambda fissure and to the protuberantia occipitalis externa, respectively. A form attribute of zero was assigned to the rest of the points. Extending the Euclidean coordinates with a form attribute coordinate, two sets of four‐dimensional points were defined:

(1) $$\;p_{m}\left(x_{m},y_{m},z_{m},a_{m}\right)\;\epsilon \;\left\{P\right\}\;\epsilon \;{\mathbb{R}}^{3}\times {\mathbb{N}}_{0}q_{n}\left(x_{n},y_{n},z_{n},a_{n}\right)\;\epsilon \;\left\{Q\right\}\;\epsilon \;{\mathbb{R}}^{3}\times {\mathbb{N}}_{0}\;$$

In Equation 1, ***{P}*** is the fixed dataset, containing M points and ***{Q}*** is the moving dataset, containing N points, respectively. The Euclidean coordinates ***x***, ***y*,** and ***z*** belong to the real coordinate space **ℝ** ^**3**^, while the form attributes ***a*** belong to the space of non‐negative integers **ℕ**_**0**_. The points are defined as vectors in the four‐dimensional space, modeled as **ℝ** ^**3**^ × **ℕ**_**0**_. A weighted distance between two points, ***p***_***m***_ and ***q***_***n***_ is formulated as

(2) $${\text{dist}}_{w}\left(p_{m},q_{n}\right)=\sqrt{{\left(x_{m}-x_{n}\right)}^{2}+{\left(y_{m}-y_{n}\right)}^{2}+{\left(z_{m}-z_{n}\right)}^{2}+w{\left(a_{m}-a_{n}\right)}^{2}}.\;$$

In Equation 2, ***w*** is a weighting factor, chosen to be a very large real number (eg, 10^9^). Thus, the weighted distances between points with equal form attributes result in the Euclidean distance between the points, while those between points with different form attributes result in very large positive real numbers. Thus, only point pairs with equal form attributes qualify in the search for the closest point. The shortest distance between a point ***q***_***n***_, from the moving dataset ***{Q}*** and the fixed dataset ***{P}*** is formulated as

(3) $$d_{n}∶=\min_{m=1\ldots M}{\text{dist}}_{w}\left(p_{m},q_{n}\right)=∶f_{1}\left(x_{n},y_{n},z_{n}\right).$$

The shortest distance (3) is defined in the discrete domain of the points in the moving dataset, where ***x***_***n***_, ***y***_***n***_, and ***z***_***n***_ are Euclidean coordinates. The minimization of the sum of squared distances, which is the generic metric by surface registration, would lead to a correct alignment of the datasets. Assuming that the points in the moving dataset are attracted by elastic forces to their closest counterparts in the fixed dataset, Hook's law^50^ can be applied to express the potential energy of a point as ***E***_***n***_ ***= kd***_***n***_^***2***^. Assuming a spring constant ***k = 1***, the total potential energy of the system in the initial position is

(4) $$E∶=\;\sum_{n=1\ldots N}{d_{n}}^{2}=∶f_{2}\left(R,t\right).$$

Expression 4 equals the absolute work done by the elastic forces by bringing the system into equilibrium. It has six degrees of freedom and is a function of the transformation parameters, where ***R*** is a rotation matrix, corresponding to rotation of the moving dataset around the origin of the coordinate system and ***t*** is a translation vector. During the optimization process only, the energy function was re‐parameterized for rotation around the instant center of rotation^50^ of the moving dataset. Minimization with a gradient‐based optimizer depends on the continuity of the minimized function.^41^ The Gaussian function is a well‐known smoothing filter kernel for step functions and a low‐pass filter. It is shown in Boughorbel et al^41^ that, after a convolution with a Gaussian kernel, a formulation of type (4) can be represented as a sum of Gaussians of the type ***exp(−d***_***n***_^***2***^ ***/σ*** ^***2***^ ***)***, aiming at better continuity and differentiability. This leads to a Gaussian scale space^31^ representation of the energy function, where the scale is the variance of the Gaussian filter, equal to the square root of the Gaussian aperture ***σ***. The computation of the partial derivatives is facilitated by the commutativity of the convolution operator and differentiation by convolution.^31^ Using development in a Taylor series, it is further shown in Boughorbel et al^41^ that for small displacements from the registered position:

(5) $$\exp\left(-\frac{{d_{n}}^{2}}{\sigma^{2}}\right)\approx 1-\frac{{d_{n}}^{2}}{\sigma^{2}}.\;$$

By proper scaling of the coordinates, through tuning the Gaussian aperture ***σ***, the range of convexity and differentiability of Equation 5 can be extended.^41^ Expressing ***d***_***n***_^***2***^ from Equation 5, the squared closest distance becomes

(6) $${d_{n}}^{2}\approx \sigma^{2}\left[1-\exp\left(-,\frac{{d_{n}}^{2}}{\sigma^{2}}\right)\right].\;$$

The real positive constant ***σ*** ^***2***^ in front of the brackets in Equation 6 can be omitted without loss of generality and influence on the convergence properties. Then, a Gaussian energy function, expressing the total potential energy of the system, is formulated as

(7) $$E_{\sigma }∶=\sum_{n=1\ldots N}\left[1-\exp\left(-\frac{{d_{n}}^{2}}{\sigma^{2}}\right)\right].$$

Our registration method is based on Boughorbel et al,^41^ however using a binary energy function, considering matches between equally attributed points only. In Boughorbel et al,^41^ the Euclidean distance was modified through the addition of the distance between the associated vectors of form attributes, and a wider basin of convergence was sought in increasing the Gaussian aperture. We have incorporated the attribute information in the computation of the closest distances between the points in Equations 2 and 3. Thus, the point matching gains a binary character, where a point‐pair with different form attributes is rejected directly. The Gaussian aperture was experimentally set to ***σ*** = 10, which proved to be the optimal scaling of the point coordinates.

The function, defining the value of the Gaussian potential energy in the discrete domain of the points in the moving dataset, is

(8) $$U\left(n\right)∶=1-\exp\left(-\frac{{d_{n}}^{2}}{\sigma^{2}}\right).\;$$

Respectively, the total potential energy of the system is

(9) $$E_{\sigma }=\sum_{n=1\ldots N}U\left(n\right).\;$$

Applying linear filtering on the potential field,^51^ defined in Equation 8, its gradient is computed as

(10) $$\vec{\mathrm{grad}}\left[U\left(n\right)\right]=\vec{\nabla }U\left(n\right)=\left[\frac{\partial U\left(n\right)}{\partial x},\frac{\partial U\left(n\right)}{\partial y},\frac{\partial U\left(n\right)}{\partial z}\right].\;$$

Equation 10 defines a vector field at the points in the moving dataset. The negative gradients are interpreted as forces of attraction, tending to fit the moving dataset onto the fixed dataset, expressed through the vector function:

(11) $$\vec{F}\left(n\right)=-\vec{\nabla }U\left(n\right).\;$$

The curl^52^ of the vector force field is computed as

(12) $$\vec{\mathrm{rot}}\left[\vec{F}\left(n\right)\right]=\vec{\nabla }\times \vec{F}\left(n\right)=\left[\frac{\partial F_{z}\left(n\right)}{\partial y}-\frac{\partial F_{y}\left(n\right)}{\partial z},\frac{\partial F_{x}\left(n\right)}{\partial z}-\frac{\partial F_{z}\left(n\right)}{\partial x},\frac{\partial F_{y}\left(n\right)}{\partial x}-\frac{\partial F_{x}\left(n\right)}{\partial y}\right].$$

The symbol “**×**” in Equation 12 indicates the cross‐product of two vectors. Analysis of the vector force field between the preoperatively and the intraoperatively generated 3D‐models allows adaptation of the algorithm to the specifics of the clinical application through the depiction of the optimal center of rotation in the optimization process. A detected point with zero curl (a vortex) would gain no (rotational) velocity by the rigid motion, initiated through the vector force field. Thus, it would act as an instant center of rotation.^50^ Under the influence of force vectors, the moving dataset would tend to rotate around its instant center of rotation. The latter was used in the parameterization of the transformation for minimization of the energy function (7).

### 2.3 Implementation details

The energy function (7) was implemented in the Insight Toolkit (ITK, ver. 4.3, Insight Software Consortium, USA), by extending the C++ class itkEuclideanDistancePointMetric, used for the formulation of metric components and for the computation of the closest points between two spatial datasets. The GetValue method in the extended class was overridden, to compute the partial derivatives (the Jacobian matrix) of the energy function with respect to each transformation parameter. The extended class was instantiated as a function object (functor)^33^ and accessed by reference by the optimizer, thus complying with the modern concept in C++^53^ for avoiding object copies. The shortest distances and the corresponding point indices were stored as vectors and returned by reference by the overridden method.

The quasi‐Newton method^33^, ^41^ was preferred to other gradient‐based optimization methods, due to its efficient function evaluation at various positions in the parameter space, achieved through an approximation of the Hessian matrix (second‐order partial derivatives).^9^, ^33^ Further, a “backtracking” strategy^33^ was applied in determining the correct step‐length in the gradient (Newtonian) direction, thus enabling a “stepping‐out” of local minima.

Surface registration was implemented as a plug‐in for the open‐source software for data visualization ParaView (ver. 5.3, Kitware Inc., Clifton Park, New York, USA). It features pipelined data‐processing and extensible architecture, inherited from VTK. ParaView was rebuilt from source code on the local 64‐bit system (Intel Core i7‐4770 at 3.40 GHz, 8 GB RAM, Windows 7), the registration algorithm was compiled as a shared library and integrated into the graphical user interface through an XML‐descriptor. On start‐up, the plug‐in displays a dialogue box for selection of the fixed and the moving datasets by the user.

The properties of continuity and convexity of the energy function (7) were evaluated in surface plots of the potential energy. Registrations on the skull phantom were compared with the GF algorithm.^41^ The same optimization routine was used for both methods; however, in ours it was assisted by the result from the VFA, and all rotations (during optimization only) were relative to the instant center of rotation of the moving dataset, while in GF those were relative to the origin of the coordinate system. The registration times were measured for varying number of points in the datasets and different precision levels. The registration error was measured at target points in clinically relevant areas on the anterior skull and visualized intuitively on a color‐coded distance map.

## 3 RESULTS

### 3.1 Computation of the vector force field and the curl

**Figure 4:**
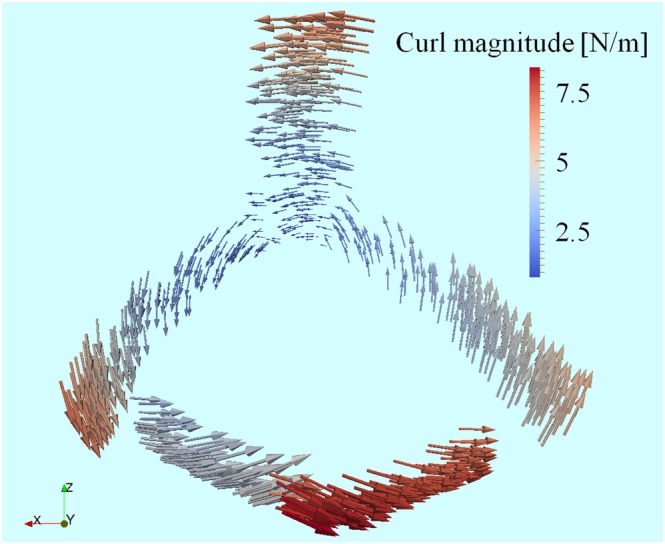
A vector force field, defined by the gradients of the energy function. The direction and the size of the arrows indicate the direction and the magnitude of the gradient, respectively. The color‐coded scale indicates the curl magnitude. (visualization: ParaView)

For visualization of the vector force field between the registration datasets, two 3D models of the occiput, generated through segmentation of CT data of the skull phantom, were manually marked with form attributes at the sutures of the Lambda fissure and the protuberantia occipitalis externa. One of them (the moving dataset) was rotationally displaced from the other one (fixed dataset). The potential function (8) and the gradient (10) were computed for all points with form attributes in the moving dataset. The vector field, defined by the gradient, is visualized in Figure 4. The arrows point in the direction of the gradient, and their sizes indicate the gradient magnitude. The gradient vectors are color‐coded with the magnitude of the curl (12) in a scale, ranging from green to red. The orientations of the gradient vectors and the curl magnitude indicate a rotational vector field. The curl magnitude tends to be zero at the mass center of the moving dataset.

**Figure 5:**
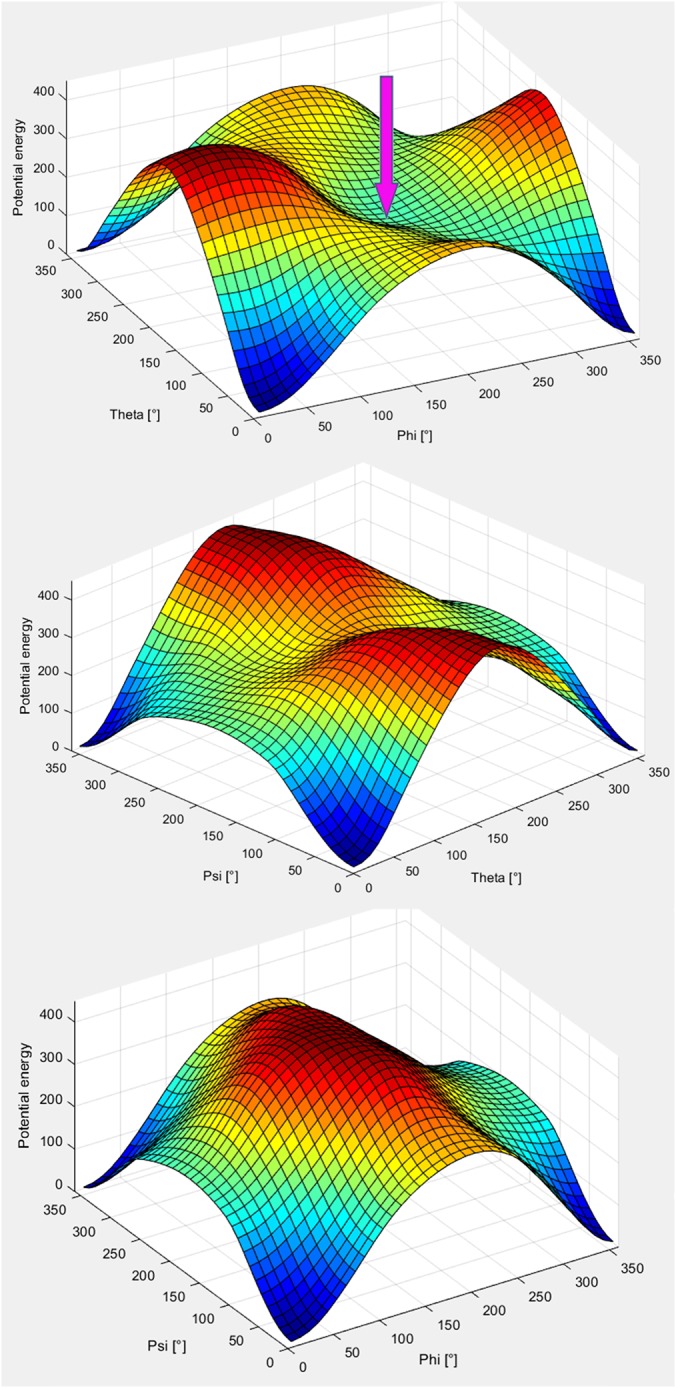
Surface plots, characterizing the energy function by rotation of the moving dataset in the domains Φ‐Θ (top), Θ‐Ψ (middle), and Φ‐Ψ (bottom) in the range [0°; 360°], starting from aligned position. The height and the color (from blue to red) of the plots indicate the value of the potential energy. The arrow in the top plot indicates a local minimum at Φ = Θ = 180°. (visualization: MatLab)

The energy function (7) is visualized in Matlab (R13, The MathWorks, Natick, MA, USA) as surface plots of the potential energy (Figure 5) in the 2D domains of all pairs of rotation parameters. It is characterized by a general convexity in the entire domain of the rotation parameters. Local minima were not completely excluded, such as the one, found at Φ = Θ = 180° (first plot in Figure 5, pointed by the arrow); however, they did not influence the overall convergence rate. Even starting from a local minimum leads to global convergence due to the “backtracking”^33^ technique of the optimizer.

### 3.2 Registration

Registrations from arbitrary initial positions (including the local minimum of E_σ_, Figure 5), spanning the entire parameter space, completed with residual error 0 (value of the energy function after convergence of the optimizer) on two identical models of the skull phantom from CT segmentation (Figure 3). Registrations of the model from CT segmentation and the model from A‐mode ultrasound scanning, containing 411 and 315 spatial points with marked form attributes, respectively, completed successfully from all initial positions with residual errors in the range 0.81 to 0.85.

**Figure 6:**
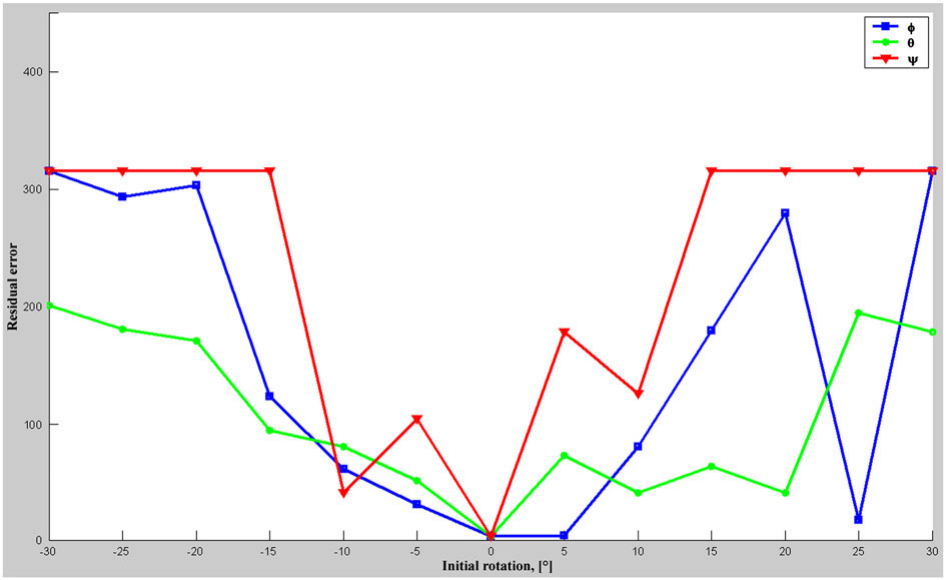
Basins of convergence of the GF algorithm by the registration of the 3D‐models (CT‐data and A‐mode ultrasound acquisition). The initial rotations around the principal axes x (angle Φ), y (angle Θ), and z (angle Ψ) are shown along the horizontal axis in the figure. The residual error of the energy function after convergence of the optimizer is shown along the vertical axis

Registrations of the datasets (CT and A‐mode ultrasound, containing 411 and 315 spatial points, respectively) with the original formulation of the GF algorithm^41^ converged globally from roughly aligned initial positions only and failed to reach global convergence from such, deviating more than ±30° from the optimal alignment. Figure 6 shows the basins of convergence of the algorithm, started at initial rotations of the moving dataset (A‐mode ultrasound) in steps of 5° around each of the principal axes. None of the registrations achieved alignment by initial rotations greater than ±30°. The figure shows that the GF has a limited convergence angle of ±10° (angle Ψ, red line).

The initial positions, listed in Table 1, converged to local minima of the GF algorithm (original formulation). Starting from the same initial positions, VFA achieved correct registrations. Table 1 contains the transformation parameters in the initial positions, the initial value of the energy function, the number of iterations of GF, the time consumed by GF, the residual error after registration with GF, the number of iterations of VFA, and the time consumed by VFA. Initial positions 5 to 8 correspond to local minima of the GF energy function. On the average, a registration with VFA needed 105.62 iterations and 56.37 seconds to converge globally, with the average time of 0.53 seconds per iteration. The residual error of the energy function corresponds to Euclidean distance, transformed to scale space, after convolution with a Gaussian kernel. In the neighborhood of the registered position, it approximates a metric in [mm] (Equation 5). For the sake of correctness, it is left unitless in the table.

**Table 1:** Registrations with VFA and comparison to the GF algorithm

Initial Position							E_σ_ (init.)	It. GF	T. GF [s]	Resid. Error GF	It. VFA	T. VFA [s]
Index	R_x_ [°]	R_y_ [°]	R_z_ [°]	X [mm]	Y [mm]	Z [mm]						
1.	38	−5	−160	0.06	0.04	0	309.72	68	368	128	115	57
2.	30	0	0	100	100	0	294.65	64	295	117.75	98	55
3.	60	0	0	0	0	0	175.44	70	329	117.75	98	53
4.	14	177	176	0	16.54	−33	312.92	44	292	117.75	96	55
5.	−39	1	−179	1.05	−143	−51	74.46	82	294	74.46	117	56
6.	0	0	−179	9.24	−165	13	60.93	39	228	60.93	117	66
7.	8	8	−177	11.79	−168	25	55.63	72	384	55.63	98	55
8.	0	−12	177	1.29	−163.6	8.08	65.63	45	231	65.63	106	54

Registrations, using VFA, were performed with datasets of a CT segmentation and an A‐mode ultrasound scan of the skull phantom, with varying numbers of points with assigned form attributes. Table 2 contains the number of iterations and the times, needed for reaching a visually correct alignment (coarse registration) and for reaching the global minimum of the energy function (fine registration). All registrations were started from initial position 4 in Table 1.

**Table 2:** Dependence of the registration times (using VFA) on the number of points

Fixed Points (CT)	Moving Points (A‐Mode US)	Iterations (Coarse)	Time (Coarse) [s]	Residual Error (Coarse)	Iterations (Fine)	Time (Fine) [s]	Residual Error (Fine)
1432	1155	24	133	2.42	86	549	1.25
1432	315	23	39	1.4	35	66	0.83
1002	315	25	30	1.43	39	50	0.81
739	315	28	31	1.38	44	42	0.81
411	315	24	12	1.48	96	55	0.85

**Figure 7:**
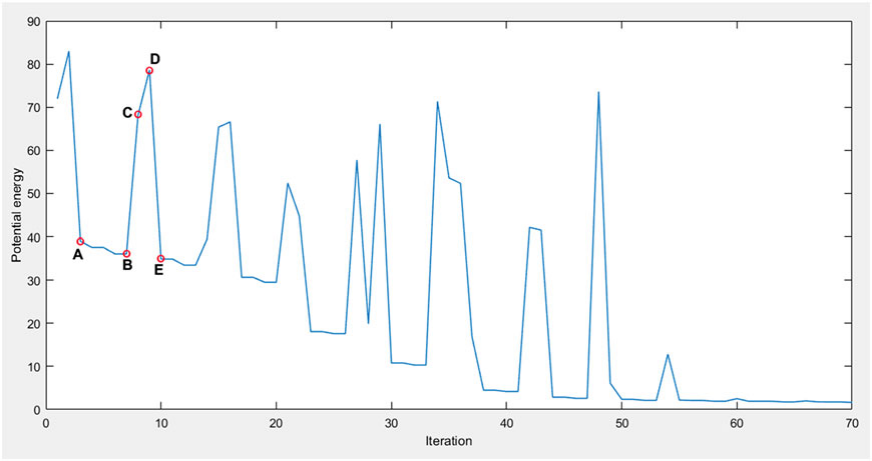
Minimization of the energy function with the quasi‐Newton method. The x‐axis shows the number of iterations of the line‐search (a sub‐routine of the quasi‐Newton optimizer). The y‐axis shows the value of the energy function

Minimization of the energy function (7) with the quasi‐Newton method for datasets with 411 and 315 spatial points is shown in Figure 7. In the waveform section, starting in position A (−7°, 1°, 14°, 13 mm, −170 mm, 57 mm), the gradient minimization reaches a local minimum in position B (−7°, 1°, 14°, 13 mm, −171 mm, 57 mm). There, the gradient is computed anew. The direction and the absolute value of the gradient provide the next Newtonian step. Taking the full Newtonian step leads to a maximum in position C. After finding no further minima, the next Newtonian step leads to a new maximum in position D. Further minima are sought through “backtracking”^33^ in the negative gradient direction, until a new minimum in position E (−21°, 26°, 11°, 27 mm, −149 mm, 78 mm) is found. The optimization continues until reaching the global minimum in the registered position (−13°, 176°, 175°, 0 mm, 24 mm, −27 mm).

### 3.3 Registration accuracy

**Figure 8:**
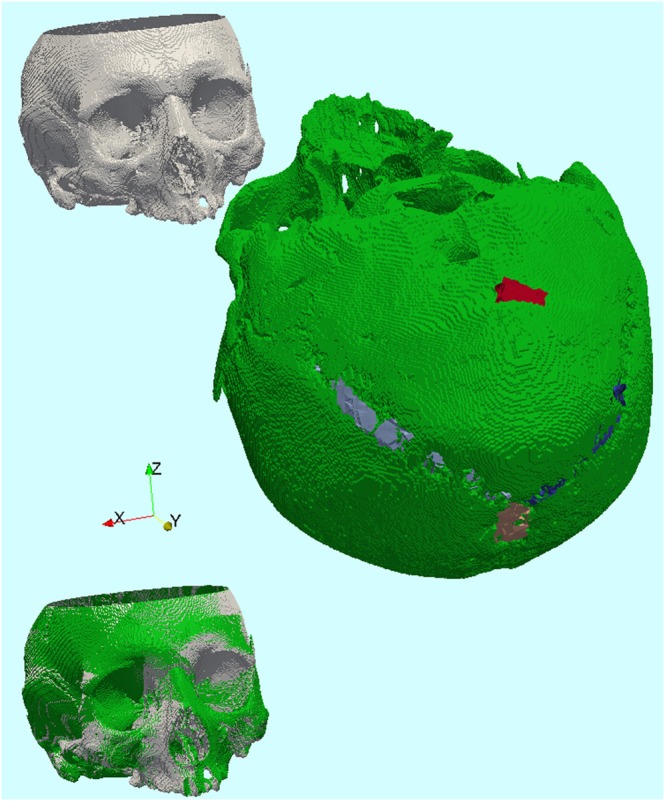
Registration of a bony skull phantom. Above: Initial position, below: Registered position. The marked form attributes in the A‐mode ultrasound acquisition are visible in the occipital area (light blue, dark blue, and beige: Lambda‐fissure sutures, red: protuberantia occipitalis externa). The coloring of the models (white: fixed, green: moving) provides intuitive indication for the clinical accuracy in the registered position. (visualization: ParaView)

For qualitative evaluation of the clinical accuracy, the registration with the proposed method is visualized in Figure 8. Two 3D models from CT segmentation of the skull phantom are in initial position 4 from Table 1 (translation along y‐axis is increased with 400 mm for better visibility, Figure 8—upper part). The model on the left has been assigned form attributes to the anatomical structures of the occiput (as already shown in Figure 3). The model on the right has been previously registered to the ultrasound dataset (form attributes are also assigned), through minimization of the energy function, reaching the global minimum of 0.81. In the lower part of Figure 8, the two 3D models are aligned through registration between the attributed points in the CT segmentation and in the ultrasound dataset. The coloring of the aligned models highlights the positional discrepancy, thus providing an intuitive indication of the registration error.

**Figure 9:**
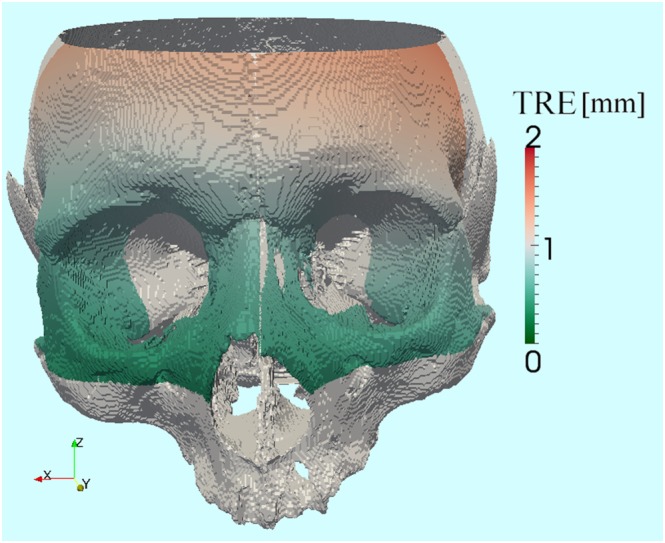
Quantitative evaluation of the clinical accuracy on the anterior skull. The color scale indicates the target registration error (TRE), measured between corresponding target points in the fixed and in the moving dataset after registration. (visualization: ParaView)

For a quantitative evaluation of the clinical accuracy (registration shown in Figure 8), the TRE^37^, ^38^ was computed for a set of target points on the anterior skull and visualized on a color‐coded map, overlaid with the 3D model of the skull phantom (Figure 9). The color scale varies from green to red with error values in the range [0.3 mm; 1.5 mm]. Sub‐millimetric accuracy has been achieved in clinically relevant ENT‐surgical areas, such as the paranasal sinuses, the frontal sinus, and the orbital cavities.

## 4 DISCUSSION

The authors are aware that the validation of this method was carried out in laboratory conditions and not in the operating room on a real patient. In a clinical application, the ultrasound waves would have to pass through a variety of tissues like dermis, epidermis, fatty tissues, tendons, and muscles with different acoustic impedances, leading to additional signal attenuation and loss of amplitude. Despite the similar speed of sound in PTFE and gel, the difference in the acoustic impedances leads to partial signal reflection and loss of intensity. As shown in Diakov et al^48^ with cadaver measurements through skin and short hair, the number of correctly measured points is reduced by approximately 30%. The artifacts are filtered out from the triangulated mesh through thresholding the triangle circumference, which does not influence registration accuracy.

The intraoperative data acquisition time is 0.125 seconds per spatial point. The generation of a moving dataset with 3000 spatial points (where 315 points are consecutively marked with form attributes) requires about 6 minutes, representing a notable interruption of the surgical workflow. Ways for reduction of the acquisition time (by half) could be sought in the implementation of continuous rotation of the sensor positioning device. Further, utilizing a priori knowledge about patient's orientation on the operating table could allow the scan of particular anatomical parts of the occiput, thus reducing the total size of the dataset.

Manual marking of form attributes makes the method semi‐automatic, and the cognitive load on the surgeon would be a potential drawback for its translation to clinical use. The procedure currently requires 8 to 9 minutes (6 minutes: intraoperative acquisition +1‐2 minutes: feature recognition and marking +1 minute: registration) to complete. A seamless workflow could be facilitated through the integration of an intuitive graphical user interface and a touch screen. The duration of the registration could be further reduced by the employment of next generation computers, parallel processing, and execution on the graphics processing unit. However, as a proof‐of‐concept, the performance of a prototype system seems acceptable.

Additional challenges would be related to the mounting of the registration device in the limited space under the operating table, which would be hindered by the presence of rotating parts near the operation field. On the other hand, A‐mode ultrasound is the optimal solution for acquisition of bone surface data, making it suitable for surface registration and automated tracking of the patient. Our registration method was validated by example for the specific application in navigated ENT surgery and can be applied on spatial data from any other modality in different surgical domains.

The convexity of the cost function in the entire parameter space was achieved through utilization of global shape information and the geometrical properties of the skull anatomy. The quality of the 3D models is important for a successful registration. The form attributes must be evenly distributed, with respect to the principal axes of the surface model, for the proper operation of the method. This condition is favored by the geometric properties of the Lambda fissure and protuberantia occipitalis externa. Registration, using the structures of the Lambda fissure only, completes successfully as well. By marking of the form attributes, a coarse outlining of the described anatomical areas yields the mentioned accuracy and no punctual precision is required from the operator. Noise is acceptable to a certain extent, as long as it is not compromising the models.

The parameterization of the transformation enables decoupling of rotation from translation and is critical for the convergence to the global minimum. A minimization search in a reduced transformation parameter space is also used by the GoICP algorithm,^15^ where global convergence is achieved through a branching and nesting strategy. The correct bounding (computed for pure rotation) is decisive for convergence.

GoICP,^15^ like the classical ICP,^5^ minimizes a cost function in Euclidean space. We performed minimization in scale space, where the spatial distances were robustified through the addition of form attributes. The convexity of the binary energy function in scale space facilitates optimization, while in the neighborhood of the registered position its values approximate Euclidean distance.

As the registration datasets are acquired on the posterior skull, the registration error would tend to increase in the direction of surgical areas on the anterior skull, due to a lever effect.^38^ Further validations, using different imaging modalities (eg, 3D‐reconstruction from a video stream) on other anatomical areas, such as patient's face, are foreseen.

As already mentioned, the purpose of this contribution is to show the feasibility of the method, allowing a unique identification of the global minimum for intraoperative registration of patient's surface data to their preoperative radiological datasets.

## 5 CONCLUSION

An innovative approach for surface registration was successfully validated on an experimental test bed with mechanically positioned A‐mode ultrasound. It proved to be suitable for clinical application in navigated ENT surgery and to be generalized over other surgical and imaging domains. The automatic and reliable patient registration equipped with intuitive guiding means is assistive to the surgeon and facilitates treatment quality.
